# Interleukin-6 deficiency exacerbates Huntington’s disease model phenotypes

**DOI:** 10.1186/s13024-020-00379-3

**Published:** 2020-05-24

**Authors:** Mary H. Wertz, S. Sebastian Pineda, Hyeseung Lee, Ruth Kulicke, Manolis Kellis, Myriam Heiman

**Affiliations:** 1 Department of Brain and Cognitive Sciences, MIT, Cambridge, MA 02139 USA; 2 Picower Institute for Learning and Memory, Cambridge, MA 02139 USA; 3 Broad Institute of MIT and Harvard, Cambridge, MA 02142 USA; 4 MIT Computer Science and Artificial Intelligence Laboratory, Cambridge, MA 02139 USA; 5 Department of Electrical Engineering and Computer Science, MIT, Cambridge, MA 02139 USA

**Keywords:** Interleukin-6, Huntington’s disease, snRNA-seq

## Abstract

Huntington’s disease (HD) is an incurable neurodegenerative disorder caused by CAG trinucleotide expansions in the *huntingtin* gene. Markers of both systemic and CNS immune activation and inflammation have been widely noted in HD and mouse models of HD. In particular, elevation of the pro-inflammatory cytokine interleukin-6 (IL-6) is the earliest reported marker of immune activation in HD, and this elevation has been suggested to contribute to HD pathogenesis. To test the hypothesis that IL-6 deficiency would be protective against the effects of mutant *huntingtin*, we generated R6/2 HD model mice that lacked IL-6. Contrary to our prediction, IL-6 deficiency exacerbated HD-model associated behavioral phenotypes. Single nuclear RNA Sequencing (snRNA-seq) analysis of striatal cell types revealed that IL-6 deficiency led to the dysregulation of various genes associated with synaptic function, as well as the BDNF receptor *Ntrk2*. These data suggest that IL-6 deficiency exacerbates the effects of mutant *huntingtin* through dysregulation of genes of known relevance to HD pathobiology in striatal neurons, and further suggest that modulation of IL-6 to a level that promotes proper regulation of genes associated with synaptic function may hold promise as an HD therapeutic target.

## Main text

The molecular mechanisms that link *huntingtin* mutation to neuronal cell death in Huntington’s disease (HD) are still not fully understood [1]. However, due to extensive reports of systemic and CNS innate immune and inflammatory activation in human HD patients and HD mouse models [2–9], it has been proposed that innate immune activation may play a role in mediating the pathogenic effects of mutant *huntingtin* (*mHTT*). Upregulation of interleukin-6 (IL-6) is the earliest reported marker of immune activation in HD, as early as 16 years before the predicted onset of clinical symptoms [10]. As the toxic properties of *mHTT* have been linked to elevation of nuclear factor-kappaB (NF-κB) activity [11], which is a potent inducer of IL-6 gene expression [12], *mHTT*-mediated upregulation of this pro-inflammatory cytokine in particular may lead to activation of neurotoxic innate immune signaling in HD. However, preclinical work in HD rodent models has suggested both a protective and negative role for IL-6 in HD [13, 14].

**Fig. 1:**
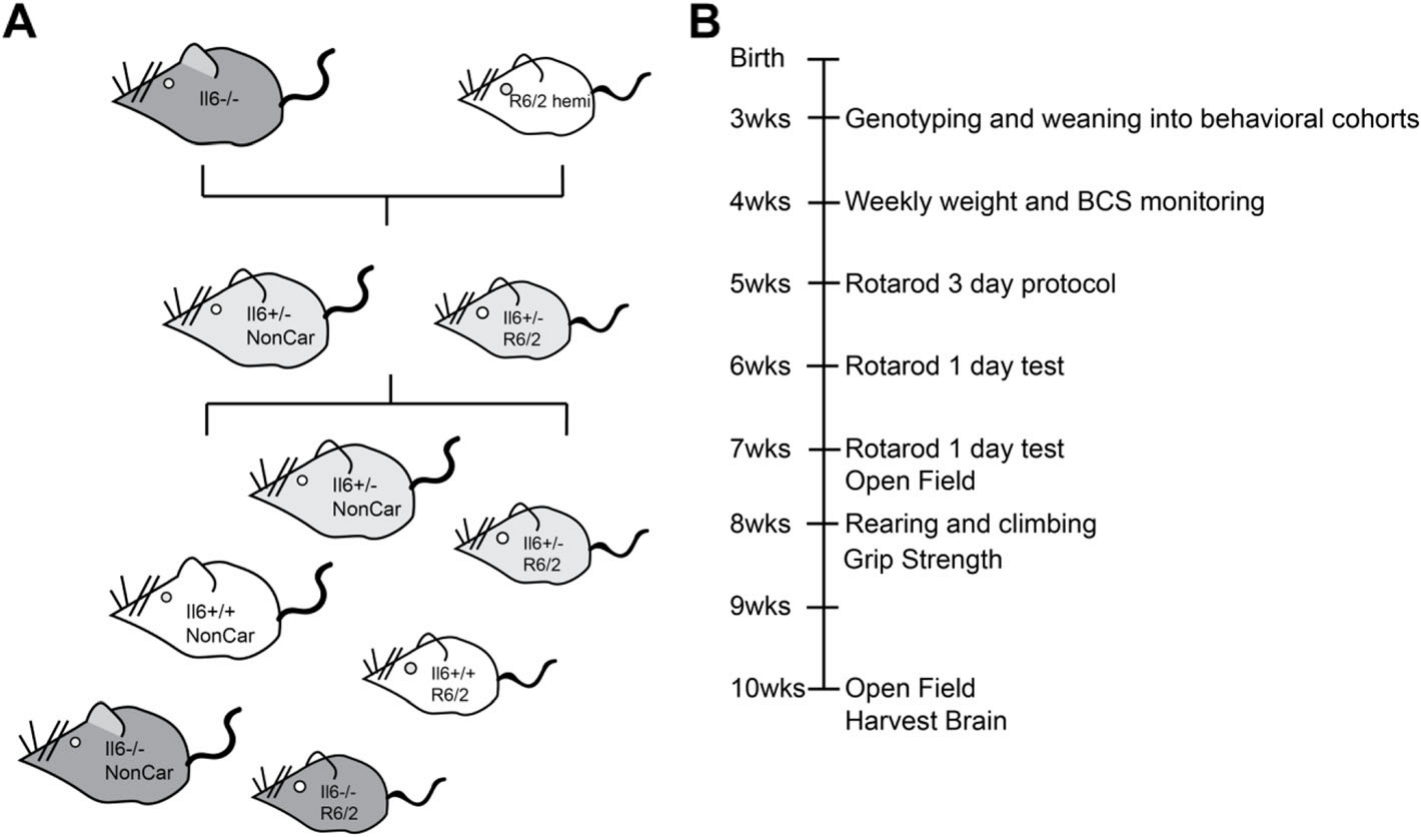
Genetic knockout of IL-6 in the R6/2 HD model. **a**. Schematic of mouse breeding and genotypes used for the study. All R6/2 (exon1 *mHTT*) HD model mice in this study were hemizygous (hemi) carriers of the R6/2 transgene. F1 heterozygous mice were used for breeding to obtain F2 mice used for behavioral and biochemical analyses. **b.** Timeline of behavioral studies in the IL-6_KO x WT and R6/2 animals. Due to advancement of HD model phenotypes, animals were harvested at 10 weeks of age after open field testing and fresh frozen striatal tissue was dissected and used for snRNA-seq analysis

**Fig. 2:**
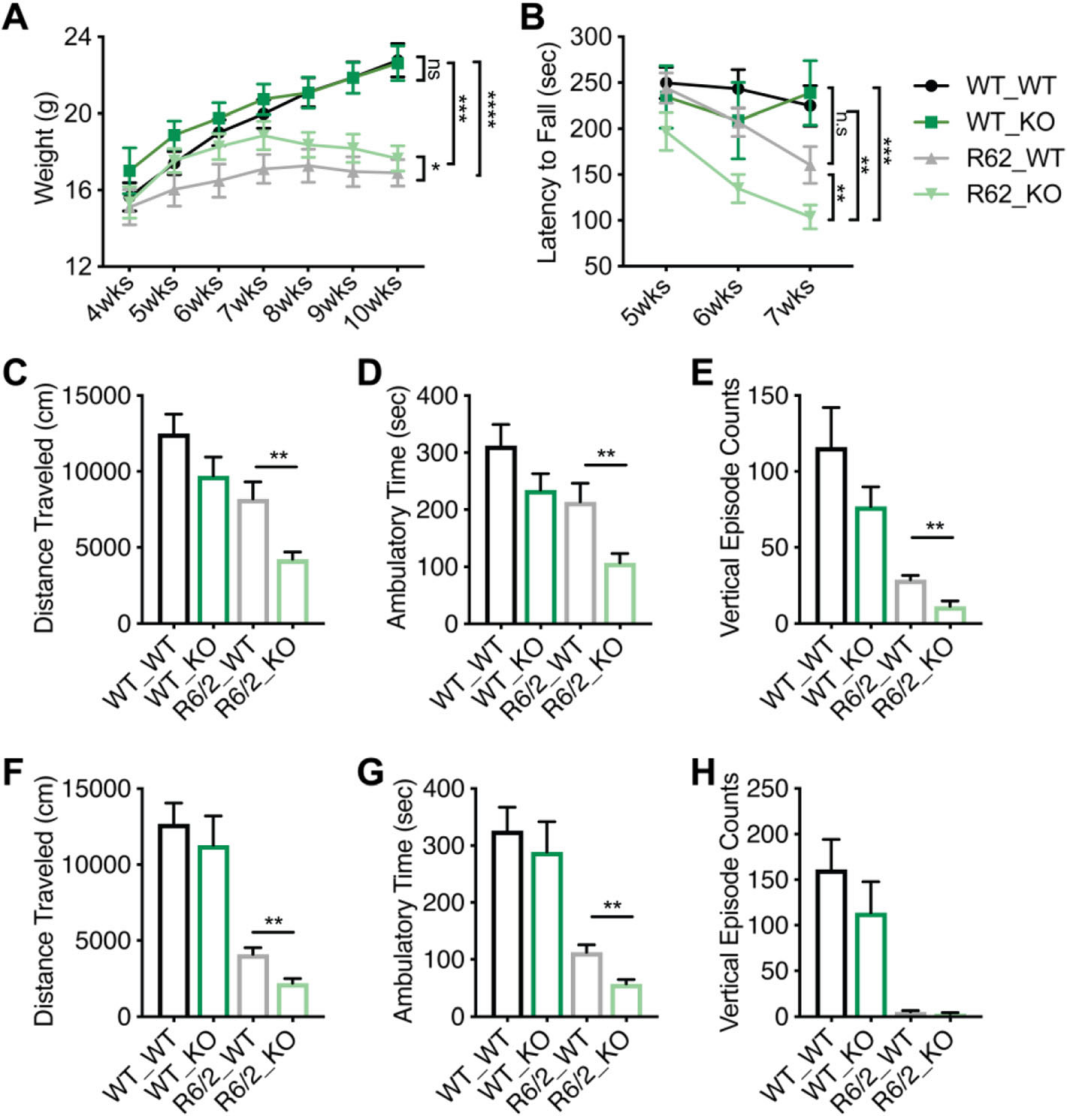
IL-6 deficiency exacerbates the R6/2 HD model behavioral phenotype. Experimental groups: WT_WT: R6/2 non-carrier and IL-6+/+; WT_KO: R6/2 non-carrier and IL-6−/−; R6/2_WT: R6/2 hemizygous carrier and IL-6+/+; R6/2_KO: R6/2 hemizygous carrier and IL-6−/−. **a.** Body weight measurements over time show that R6/2_WT mice lose more weight than the R6/2_KO mice as compared to WT_WT or WT_KO controls. Mixed effects model (restricted maximum likelihood REML), *p* < 0.0001, Tukey’s multiple comparison *p* = 0.027 (*), *p* = 0.0005 (***), *p* < 0.00001 (****). Number of animals per group: WT_WT (*n* = 10), WT_KO (*n* = 5), R6/2_WT (*n* = 10), R6/2_KO (*n* = 14). **b.** R6/2_KO mice perform more poorly than R6/2_WT mice on the rotarod assay at 5–7 weeks of age. Number of animals per group: WT_WT (*n* = 10), WT_KO (*n* = 5), R6/2_WT (*n* = 10), R6/2_KO (*n* = 13). Mixed effects model (restricted maximum likelihood REML), *p* < 0.05, Tukey’s multiple comparison *p* < 0.01 (**), *p* < 0.0001 (****). **c-e.** R6/2_KO mice demonstrate less spontaneous motor activity than R6/2_WT mice, as measured by horizontal distance traveled, ambulatory time, and vertical episodes in the open field assay at 7 weeks of age. Number of animals per group; WT_WT (*n* = 10), WT_KO (*n* = 5), R6/2_WT (*n* = 10), R6/2_KO (*n* = 12). *p* < 0.01 (**), two-tailed *t*-test. **f-h.** R6/2_KO mice demonstrate less spontaneous motor activity than R6/2_WT mice, as measured by horizontal distance traveled, ambulatory time, and vertical episodes in the open field assay at 10 weeks of age. Number of animals per group: WT_WT (*n* = 10), WT_KO (*n* = 5), R6/2_WT (*n* = 9), R6/2_KO (*n* = 11). *p* < 0.01 (**), two-tailed *t*-test. All data are represented as mean ± standard error of the mean

To investigate the role of IL-6 in protecting against or promoting the pathogenic effects of *mHTT* from early phenotypic stages, we crossed R6/2 transgenic exon 1 *mHTT* model mice [15] to mice that that were deficient in IL-6 [16] and performed behavioral assays of HD motor phenotype progression (Schematic: Fig. 1). Consistent with previous reports that IL-6 −/− mice do not demonstrate differences in their spontaneous motor activity [17], we did not observe any differences in the rotarod performance or open field and rearing and climbing behaviors between IL-6 −/− and IL-6 +/+ wild type control mice (WT, those that did not carry the R6/2 exon1 *mHTT* transgene) (Fig. 2a-h and S1A-F). And consistent with reports that IL-6 exerts anti-obesity effects in rodents [18], IL6 −/− mice that carried the R6/2 transgene had slightly higher overall weights than their IL-6 +/+ R6/2 transgenic littermates (Fig. 2a). However, IL-6 −/− mice that carried the R6/2 transgene had more severe HD-associated behavioral symptoms than their IL-6 +/+ R6/2 transgenic littermates as assessed by rotarod performance (Fig. 2b and S1A), open field assay measurements (Fig. 2c-h), and rearing and climbing activity (Fig. S1B-F). The only assay in which IL-6 −/− mice that carried the R6/2 transgene did not have more severe HD-associated symptoms than their IL-6 +/+ R6/2 transgenic littermates was in the grip strength test (Fig. S1G). And although the IL-6 −/− mice that carried the R6/2 transgene had slightly higher overall body weights than their IL-6 +/+ R6/2 transgenic littermates (Fig. 2a), there was no significant correlation between mouse body weight and performance on the rotarod test (Fig. S2). Finally, since the R6/2 model mice are usually bred and tested in a mixed strain background (CBA x C57BL/6), we performed single nucleotide polymorphism (SNP) array genotyping to assess the strain characteristics of the F2 mice used in behavioral testing. We observed no significant correlation between the percentage similarity to the congenic C57BL6 strain background and motor testing performance (Fig. S3), and thus we conclude that whatever differences in strain background that exist among the tested F2 mice do not account for the differences in behavioral testing results, and that these are rather a result of the IL-6 genotype. Thus together our data reveal that constitutive IL-6 loss exacerbates several HD-like behavioral symptoms in the R6/2 exon1 *mHTT* model.

**Fig. 3:**
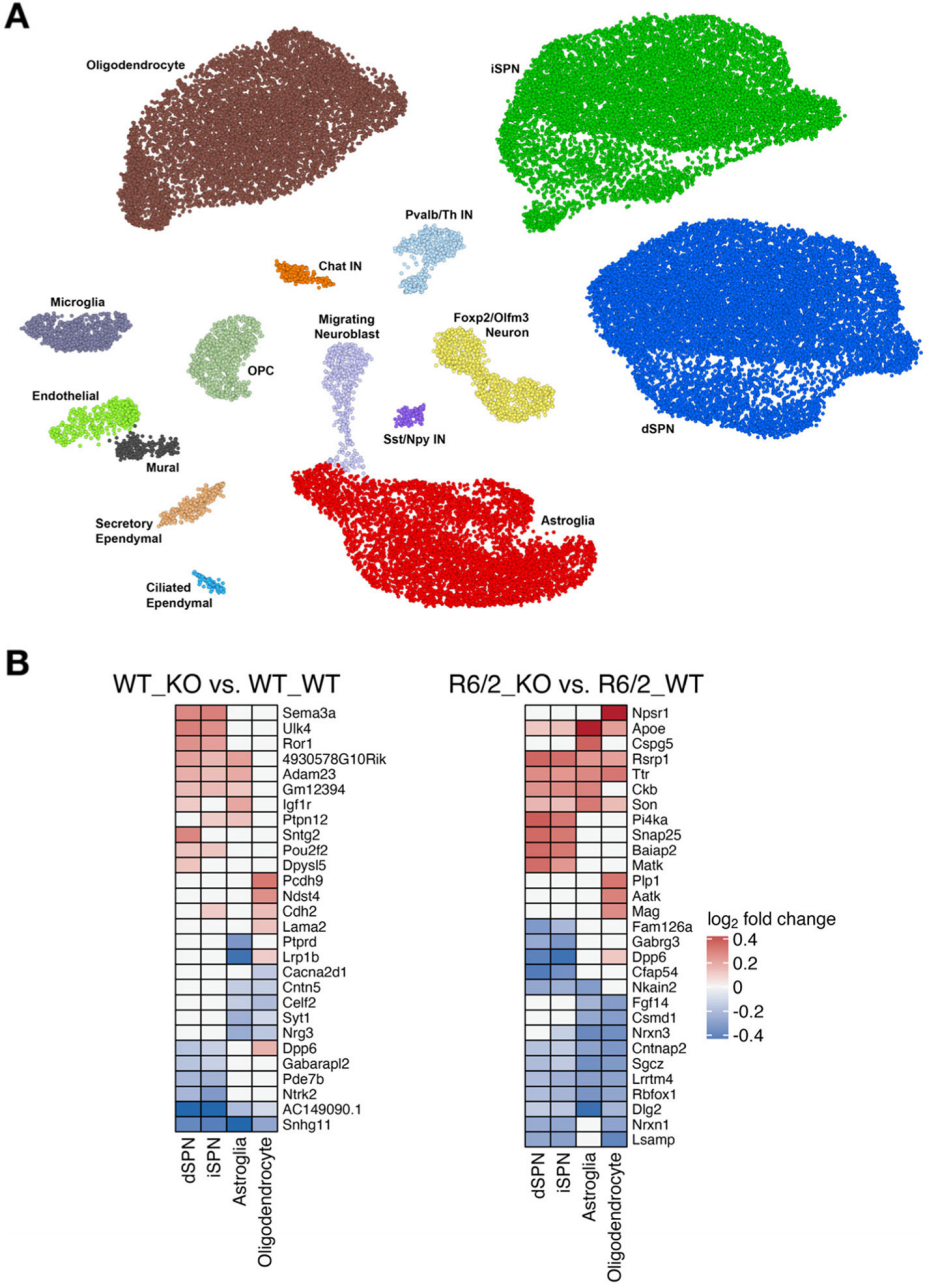
snRNA-seq from R6/2 IL-6 knockout mice reveals cell-type specific gene expression changes in striatal cell types induced by IL-6 deficiency. **a**. ACTIONet plot of striatal cell types detected by snRNA-seq. **b.** The top five most downregulated and upregulated non-mitochondrial, protein-coding genes by log_2_-fold change in the most abundant striatal cell types induced by IL-6 deficiency in WT_KO (left panel) and R6/2_KO HD model mice (right panel)

**Fig. 4:**
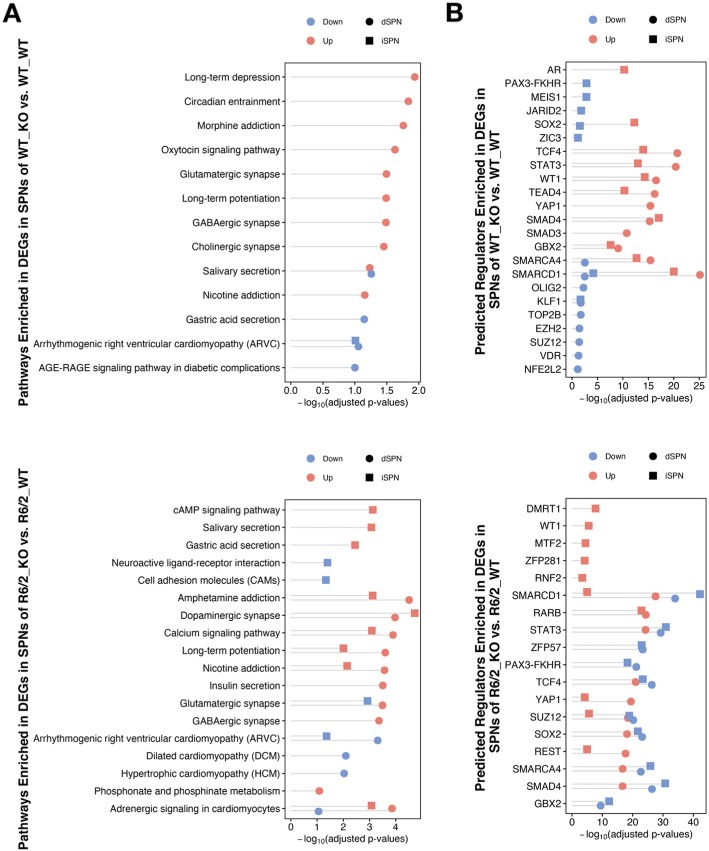
Gene pathways and predicted transcriptional regulators affected by IL-6 knockout. **a**. Enriched KEGG pathways of genes downregulated and upregulated in dSPNs and iSPNs upon IL-6 deficiency in WT_KO (top panel) and R6/2_KO HD model mice (bottom panel), represented with Fisher’s exact test –log_10_-adjusted *p*-value. **b.** Predicted transcriptional regulators, by ChEA analysis, of genes that were downregulated and upregulated in dSPNs and iSPNs upon IL-6 deficiency in WT_KO (top panel) and R6/2_KO HD model mice (bottom panel), represented with Fisher’s exact test –log_10_-adjusted *p*-value

In order to investigate the molecular basis for the aggravation of HD model phenotypes upon IL-6 KO, we performed single nuclear RNA-sequencing (snRNA-seq, *n* = 3 per group; Methods) on nuclei isolated from the striatal tissue of the same mice that were used for behavioral testing, harvested at 10 weeks of age. We used the ACTIONet framework [19] to identify major cell types in the striatum across the replicate samples in each group. Using a curated set of marker genes, we recovered major expected cell types in the striatum, including the two major types of striatal neurons, direct and indirect pathway spiny projection neurons (dSPNs and iSPNs), as well as astroglia, microglia, oligodendrocytes, oligodendrocyte precursor cells, *Chat*-expressing cholinergic interneurons, *Sst*/*Npy*-expressing GABAergic interneurons, *Pval*/*Th*-expressing GABAergic interneurons, and endothelial and mural cells of the blood brain barrier. Since the subventricular zone was included in the striatal dissection, we also recovered ciliated ependymal cells, secretory ependymal cells, and migrating neuroblasts (Fig. 3a and Methods). Additionally, we recovered a distinct *Foxp2*/*Olfm3*-expressing neuron cluster representing a distinct neuron subtype that is characterized by expression of *Olfm3*, *Foxp2*, *Adarb2*, and *Otof* that likely represents the same striatal neuronal subtype recently characterized in other studies as expressing *Otof* and *Olfm3* [20, 21]. Differential gene expression analysis of the most abundant identified cell types (dSPNs, iSPNs, astroglia, and oligodendrocytes) (Methods) revealed changes to gene expression occurring upon IL-6 KO in non-carrier control (WT) mice and in the R6/2 mice (Fig. 3b and Tables S1-S2). Pathway analysis of the differentially expressed genes revealed that among the top gene pathways altered in SPNs by IL-6 KO in both the R6/2 carrier and WT mice were various terms related to synaptic transmission, including terms related to the glutamatergic synapse and long-term potentiation (Fig. 4a). Chromatin Enrichment Analysis (ChEA) [22] for predicted regulators of these changes in SPNs revealed that *Stat3* was among the top predicted regulators of these gene expression changes in SPNs (Fig. 4b). In support of *Stat3* directly linking IL-6 deficiency to alterations in genes involved in synaptic transmission, *Stat3* is not only one of the canonical transcription factors that transduces IL-6 receptor signaling [23] but also has been shown to have a role in the regulation synaptic plasticity [24]. In addition, we noted that the brain-derived neurotrophic factor (BDNF) receptor *Ntrk2* was downregulated in WT and R6/2 SPNs that lacked IL-6, and several synaptic protein-encoding genes such as *Nrxn1*, *Dlg2*, *Cntnap2*, and *Gabrg3* were among the most downregulated genes in R6/2 SPNs that lacked IL-6 (Fig. 3b and Tables S1-S2). Although we did not recover enough microglial cells to determine with high confidence genes that were differentially expressed in striatal microglial cells upon IL-6 deficiency, analysis of gene pathways and predicted regulators of gene expression changes that were detected in striatal astroglia and oligodendrocytes did not show evidence of alteration of glial inflammatory or innate immune signaling pathways (Figs. S4). Although it is possible that glial populations in mouse models of HD may not fully recapitulate changes to gene expression that are observed in human glial populations in HD, our gene expression data suggest that IL-6 deficiency does not alter innate immune activation in an HD context, but rather may aggravate HD model phenotypes in part by dysregulation of genes related to synaptic transmission and neurotrophin signaling, two pathways that have been linked to *mHTT* pathogenesis [25, 26].

In conclusion, our data reveal that a constitutive KO of IL-6, which is normally expressed in both neurons and glia in the CNS [27], including in the striatum [28], exacerbates several HD-related behavioral phenotypes in the R6/2 exon1 *mHTT* model of HD. Combined with reports of a protective effect of IL-6 in models of traumatic brain injury [29, 30], Parkinson’s disease [31, 32], and in modulating Aβ deposition in Alzheimer’s disease models [33] it is thus possible that the clinical reports of IL-6 elevation in HD at early disease timepoints may reflect a protective rather than pathogenic alteration, and that elevation of IL-6 levels, in certain ranges and timepoints, may have therapeutic benefit in HD.

## Methods

### Animal usage

All animal experiments were conducted with the approval of the Massachusetts Institute of Technology Animal Care and Use Committee. Mice were housed with food and water provided ad libitum on a standard 12 h light/12 h dark cycle. All mice were obtained from the Jackson Laboratory (Bar Harbor, ME). R6/2 HD model mice (B6CBA-Tg (HDexon1)62Gpb/1 J, Jackson Laboratory stock #002810) were crossed with IL-6 knockout mice maintained the C57BL/6 background (B6.129S2-Il6tm1Kopf/J, Jackson Laboratory stock #002650). F1 heterozygous IL-6 −/+ x R6/2 hemizygous mice were crossed to obtain the F2 generation, which was used for experiments between 4 and 10 weeks of age. Given the two-strain mixed background of the original R6/2 breeders (CBA and C57BL/6), strain characteristics for each mouse used in behavioral testing were assayed by single nucleotide polymorphism (SNP) genotyping (Transnetyx, Cordova, TN, Full Strain Genetic Monitoring, 120 SNPs with markers on all autosomes); these data are presented in Fig. S3. Beginning at 4 weeks of age, animals were monitored weekly for body condition score and weight. Both male and female mice were used for the behavioral and biochemical analyses. Behavioral testing experiments were performed on mice at the time points outlined in Fig. 1 and listed in the *Behavioral Testing* section below, and mice were otherwise naïve to the testing or prior analyses.

### Behavioral testing

Behavioral analyses were performed on the F2 animals as previously described [34]. Behavioral testing was conducted and analyzed by an investigator blinded to genotype. Statistical testing was performed using GraphPad Prism 8 with the number of mice (*n*) per group and the statistical measures performed as reported in detail in the figure legends. Behavioral data is presented as mean ± standard error of the mean unless otherwise stipulated. Mice were identified as outliers and excluded from further analysis if they scored ±2 standard deviations from the mean on multiple behavioral tests.

Rotarod testing was performed using an accelerating rotarod (Med Associates, St. Albans, VT). Mice were trained using 3 consecutive 5 min sessions with a fixed speed of 20 rotations per minute (RPM) and gently placed back on the rotarod after each fall. Mice were given 1 min of rest between training trials. At 5 weeks of age, testing was performed on 5 consecutive days with the rotarod accelerating from 5 RPM to 40 RPM over the course of 5 min. Latency to fall was measured as the time from the beginning of a trial until the mouse fell off the rod or completed two or more passive rotations. On subsequent weeks mice were re-tested on the accelerating rotarod on a single day to assess their motor phenotype over time.

Open Field testing was performed at 7 and 10 weeks of age in 60-min sessions each week per mouse using an infrared photobeam open field chamber. The field had 16 infrared beams spaced regularly along the *x*, *y*, and *z* axes (#MED-OFAS-RSU, Med Associates, St. Albans, VT). Data was analyzed for distance traveled, time traveling, vertical activity and vertical time.

Rearing and Climbing analysis was performed by placing mice under an overturned black metal mesh pencil cup (Rolodex #82406) 4.375 in. in diameter and 5.5 in. in height. The latency until the mouse reared on hind legs and touched the mesh with its front paws as well as the latency until the mouse climbed on the mesh with all paws off of the ground were recorded. All subsequent rearing and climbing events were counted, and summed together to get the total vertical activity. Mice were given one 5 min trial at 8 weeks of age.

Grip strength was performed using a grip strength meter (Ugo Basile,Varese, Italy). Briefly. The mice were suspended by their tails and allowed to grab the measurement bar. They were then pulled away from the bar by the tail until they released the bar and the maximum force (*g*) was recorded. Each mouse was given 5 trials. Trials where the mouse failed to grasp the bar with two hands were excluded from subsequent analyses. The average maximum force of the 5 trials was used for each mouse.

### EnrichR pathway analysis

Pathway and chromatin enrichment analysis was performed using the EnrichR package [35, 36] considering only protein-coding genes. Significant pathways were identified by Fisher’s exact test with adjusted *p*-value < 0.05.

### Single nuclear (snRNA) RNA sequencing and analysis

Nuclei isolation protocol was adapted from [37]. Briefly, striata were dissected and flash frozen in liquid nitrogen. Frozen tissue was homogenized in 700 μL of homogenization buffer with a 2 mL KIMBLE Dounce tissue grinder (MilliporeSigma, Burlington MA) using 10 strokes with loose pestle followed by 10 strokes with tight pestle. Homogenized tissue was filtered through a 40 μm cell strainer and mixed with 450 μL of working solution (50% OptiPrep density gradient medium (MilliporeSigma, Burlington MA), 5 mM CaCl_2_, 3 mM Mg(CH_3_COO)_2_, 10 mM Tris HCl [pH 7.8], 0.1 mM EDTA [pH 8.0], and 1 mM β-mercaptoethanol). Nuclei were pelleted at the interface of an OptiPrep density gradient containing 750 μL of 30% OptiPrep Solution on top of 300 μL of 40% OptiPrep Solution inside a Sorenson Dolphin microcentrifuge tube (MilliporeSigma, Burlington MA) by centrifugation at 10,000 x *g* for 5 min at 4 °C using a fixed angle rotor (FA-45-24-11-Kit). The nuclear pellet was collected at the interface and washed with 2% BSA (in 1x PBS) containing 0.12 U/μL SUPERase In RNase Inhibitor. The nuclei were pelleted by centrifugation at 300 x *g* for 3 min at 4 °C using a swing-bucket rotor (S-24-11-AT). Nuclei were washed three times with 2% BSA and centrifuged under the same conditions. The nuclear pellet was re-suspended in 100 μL of 2% BSA.

Droplet-based snRNA sequencing libraries were prepared using the Chromium Single Cell 3′ Reagent Kit v3 (10x Genomics, Pleasanton CA) according to the manufacturer’s protocol and sequenced on a NovaSeq 6000 at the Broad Institute Genomics Platform. FASTQ files were aligned to the pre-mRNA annotated *Mus musculus* reference genome version GRCm38.

The R package batchelor [38], was used to correct for batch effects observed across biological replicates within each experimental group from the count matrix. Batch-corrected data was used as input to the archetypal analysis for cell type identification (ACTION) algorithm [39] to identify a set of landmark cells or ‘archetypes’, each representing a potential underlying cell state. Using ACTION-decompositions with varying numbers of archetypes, we employed our recently developed ACTION-based network (ACTIONet) framework [19] to create a multi-resolution nearest neighbor graph. ACTIONet graphs were visualized using a modified version of the stochastic gradient descent-based layout method in the uniform manifold approximation and projection (UMAP) algorithm [40]. A curated set of known cell type-specific markers was used to annotate individual cells with their expected cell type and assign a confidence score to each annotation. Network connectivity was used to correct low-confidence annotations. Multiple iterations of this process were performed to identify and prune low quality cells, such as those with ambiguous profiles resembling dissimilar cell types (generally corresponding to doublet nuclei), or cells corresponding to nodes with a low *k*-core number in the network (generally corresponding to high ambient RNA content or doublet nuclei).

Cell-wise gene counts were normalized and log-scaled using the R package scran using cell type and genotype as normalization factors. Differential gene expression analysis was performed using Wilcoxon rank-sum test with the R package presto. Genes were considered differentially expressed if they had an absolute log-fold change > 0.1 with FDR < 0.001 vs. the respective control in each statistical test.

### Statistical analysis

GraphPad Prism 8 was used to perform the statistical analysis of the behavioural testing data experiments. Specific information on the number (*n*) of values used as well as the statistical tests applied to the data can be found in the figures and/or figure legends.

## Supplementary information

**Additional file 1:** Supplementary materials.

## Data Availability

The datasets generated during and analyzed in the current study are available in the NCBI GEO repository, under accession # GSE143355.

## Abbreviations

BDNF: Brain-derived neurotrophic factor
BSA: Bovine serum albumin
cAMP: Cyclic adenosine monophosphate
ChEA: Chromatin enrichment analysis
CNS: Central nervous system
dSPN: Direct pathway spiny projection neuron
EDTA: Ethylenediaminetetraacetic acid
F1: First filial generation
F2: Second filial generation
FDR: False discovery rate
h: Hour
HD: Huntington’s disease
IL-6: Interleukin-6
iSPN: Indirect pathway spiny projection neuron
KEGG: Kyoto encyclopedia of genes and genomes
KO: Knockout
mHTT: Mutant huntingtin
min: Minute
n: Number
NF-κB: Nuclear factor-kappaB
Ntrk2: Neurotrophic receptor tyrosine kinase 2
PBS: Phosphate buffered saline
REML: Restricted maximum likelihood
RPM: Revolutions per minute
RNA: Ribonucleic acid
snRNA-Seq: single nuclear RNA sequencing
SNP: Single nucleotide polymorphism
SPN: Spiny projection neuron
Stat3: Signal transducer and activator of transcription 3
Tris: Tris (hydroxymethyl)aminomethane
UMAP: Uniform manifold approximation and projection
UMIs: Unique molecular identifiers
WT: Wild type
