# Supplementary material for: Interleukin-6 deficiency exacerbates Huntington’s disease model phenotypes
Source: Mol Neurodegener. 2020 May 24;15:29. doi: 10.1186/s13024-020-00379-3 (PMC7247164; doi:10.1186/s13024-020-00379-3)
Supplement: Supplementary file 1 — Additional file 1: Supplementary materials. [file 13024_2020_379_MOESM1_ESM.zip › 13024_2020_379_MOESM1_ESM/Wertz et al Supplement_ESM.pdf]

## SUPPLEMENTARY MATERIALS

**Table S1 (Excel File).** Single nuclear RNA-Sequencing (snRNA-seq) of cell type-specific changes to gene expression (*p*-value adj. < 0.001 and $\log_2$ fold change > 0.1 or < -0.1) in striatal cell types in wild type control vs. wild type IL-6 KO mice.

**Table S2 (Excel File).** Single nuclear RNA-Sequencing (snRNA-seq) of cell type-specific changes to gene expression (*p*-value adj. < 0.001 and $\log_2$ fold change > 0.1 or < -0.1) in striatal cell types in R6/2 HD model vs. R6/2 HD model IL-6 KO mice.

**Figure S1.** IL-6 deficiency exacerbates additional R6/2 behavioral phenotypes.

**Figure S2.** Lack of correlation between mouse body weight and mouse performance on the rotarod test at 6 weeks of age.

**Figure S3.** Lack of correlation between the genetic similarity of individual mice to the congenic C57BL/6 background and behavioral testing performance.

**Figure S4.** snRNA-seq from WT_KO and R6/2_KO mice reveals cell-type specific gene expression changes in astroglia and oligodendrocytes that are induced by IL-6 deficiency.

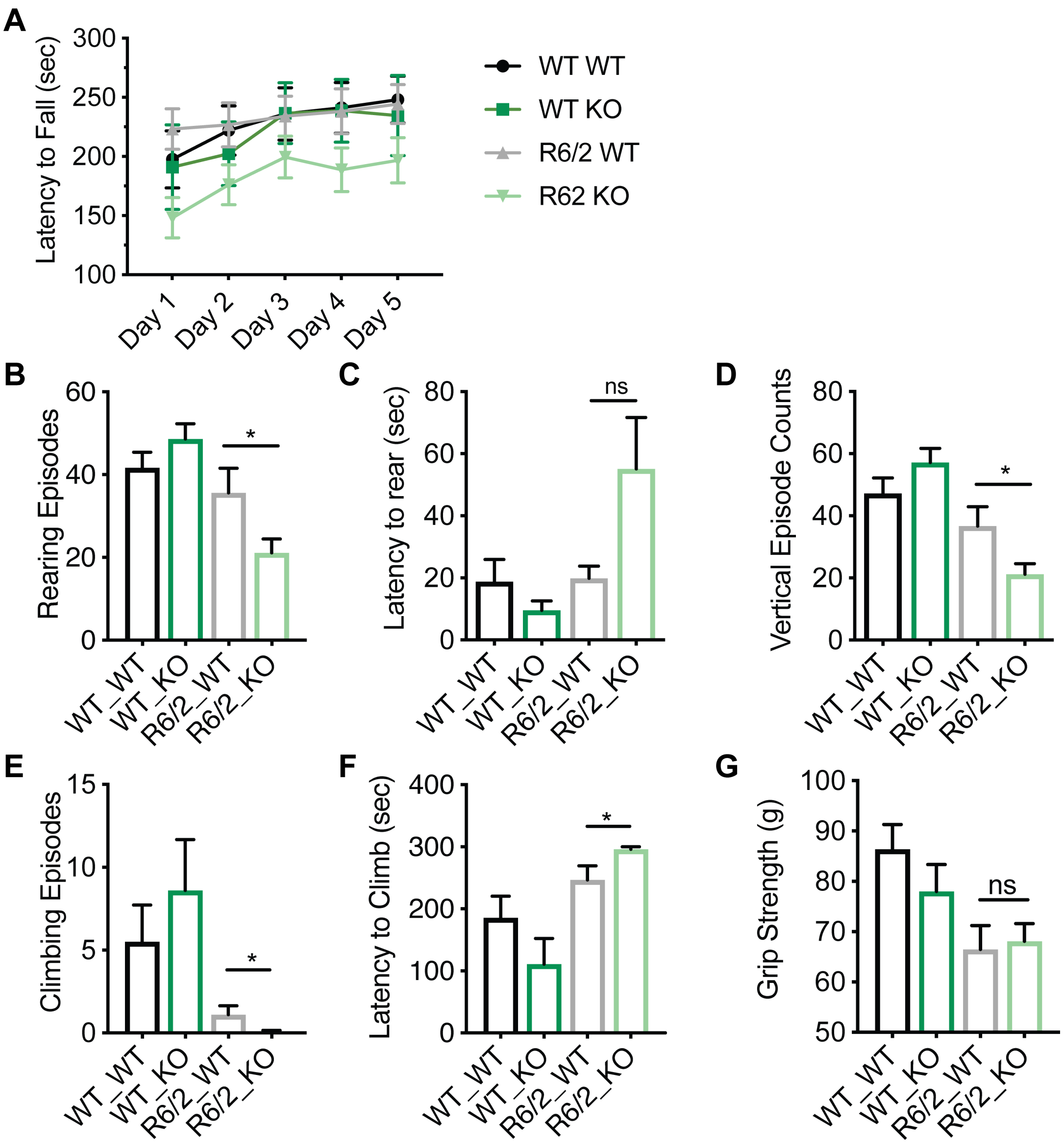


**Figure S1. IL-6 deficiency exacerbates additional R6/2 behavioral phenotypes. A.** Rotarod performance over a 5-day testing paradigm at 5 weeks of age. Number of animals per group; WT_WT (*n*=10), WT_KO (*n*=5), R6/2_WT (*n*=10), R6/2_KO (*n*=13). Two-way repeated measured ANOVA $p$ = ns., Tukey's multiple comparison $p = 0.025$ (*) only for R6/2_WT vs. R6/2_KO on day 1 of testing, indicating a difference in the ability of the IL6_KO mice to learn the motor task (further significances are seen upon further testing, Figure 2). **B-F** Rearing and

climbing testing at 8 weeks of age reveals a deficit in rearing episodes, latency to rear, climbing episodes, latency to climb and total vertical activity in the R6/2_KO as compared to R6/2_WT mice. $p < 0.05$ (*), two-tailed student's *t*-test. **G.** While both the R6/2_WT and R6/2_KO have decreased grip strength as compared to controls, there is no significant difference. All data are represented as mean ± standard error of the mean.

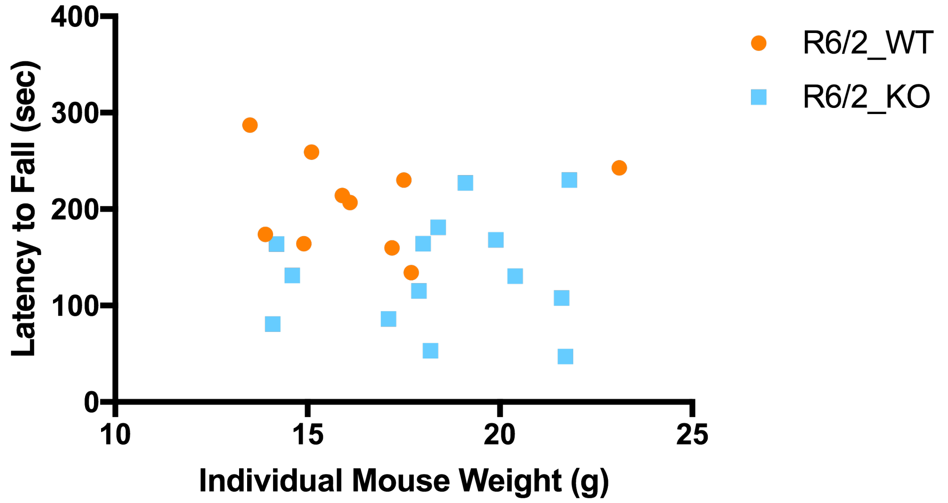


**Figure S2. Lack of correlation between mouse body weight and mouse performance on the rotarod test at 6 weeks of age.** Individual mouse data for the R6/2_WT and R6/2_KO mice at 6 weeks of age, plotting weight against latency to fall on the rotarod test. Pearson correlation coefficient for R6/2_KO $r = 0.129$, two-tailed $p = 0.660$.

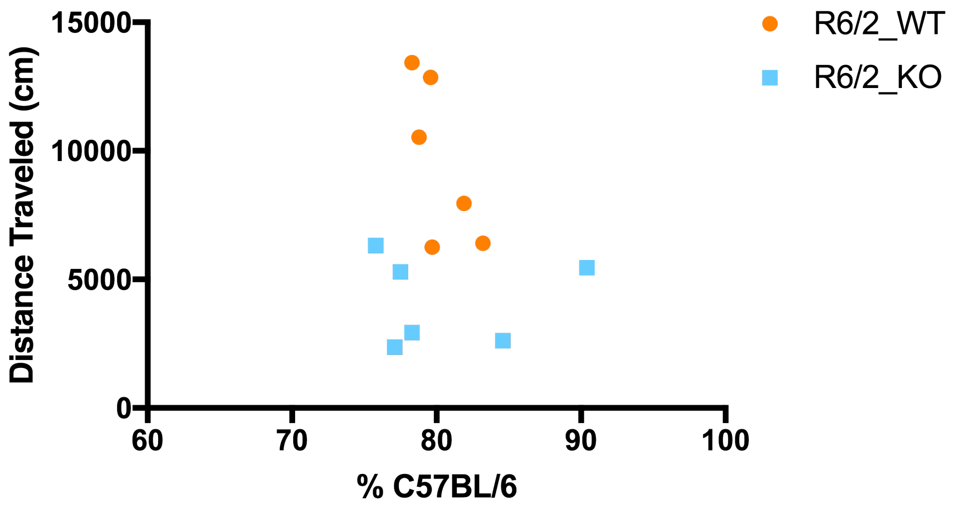


**Figure S3. Lack of correlation between the genetic similarity of individual mice to the congenic C57BL/6 background and behavioral testing performance.** Individual mouse data for the R6/2_WT and R6/2_KO mice, plotting percentage (%) similarity to the congenic C57BL/6 strain background (as reported by SNP genotyping) against horizontal distance traveled in the open field assay at 7 weeks of age (for all mice in Figure 2C for which SNP genotyping data was available). Pearson correlation coefficient for R6/2_KO $r = 0.036$, two-tailed $p = 0.946$.

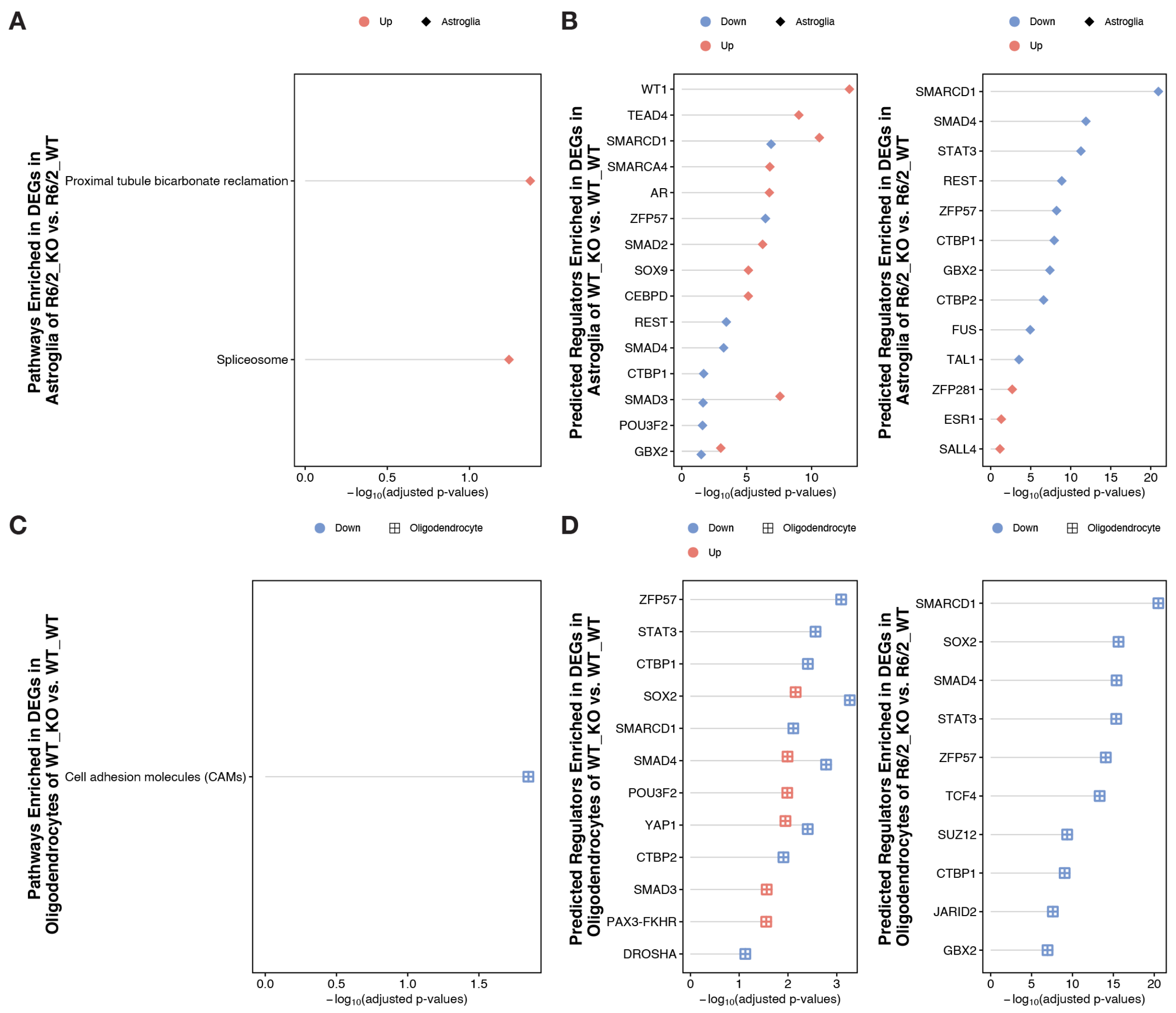


**Figure S4. snRNA-seq from WT_KO and R6/2_KO mice reveals cell-type specific gene expression changes in astroglia and oligodendrocytes that are induced by IL-6 deficiency. A.** Enriched KEGG pathways of genes upregulated in astroglia upon IL-6 KO in R6/2 mice, represented with Fisher's exact test –$\log_{10}$-adjusted *p*-value. There were no significantly enriched pathways among the astroglial downregulated genes, or in the upregulated and downregulated genes in the WT_KO vs. WT_WT comparison. **B.** Predicted transcriptional

regulators, by ChEA analysis, of genes that were downregulated and upregulated in striatal astroglia upon IL-6 KO in control mice (left panel) and R6/2 HD model mice (right panel), represented with Fisher's exact test –$\log_{10}$-adjusted *p*-value. **C.** Enriched KEGG pathways of genes downregulated in oligodendrocytes upon IL-6 KO in control mice, represented with Fisher's exact test –$\log_{10}$-adjusted *p*-value. There were no significantly enriched pathways among the oligodendrocyte upregulated genes, or in the upregulated and downregulated genes in the R6/2_KO vs. R6/2_WT comparison. **D.** Predicted transcriptional regulators, by ChEA analysis, of genes that were downregulated and upregulated in striatal oligodendrocytes upon IL-6 KO in control mice (left panel) and R6/2 HD model mice (right panel), represented with Fisher's exact test –$\log_{10}$-adjusted *p*-value.
